# Editorial: Alzheimer's Disease From a Psychiatric Perspective: Towards New Therapeutic Guidelines?

**DOI:** 10.3389/fpsyt.2021.782423

**Published:** 2021-11-04

**Authors:** Marc Fakhoury, Fabrizio Piras, Nerisa Banaj

**Affiliations:** ^1^Department of Natural Sciences, School of Arts and Sciences, Lebanese American University, Beirut, Lebanon; ^2^Laboratory of Neuropsychiatry, IRCCS Santa Lucia Foundation, Rome, Italy

**Keywords:** Alzheimer's disease, aging, cognitive functions, dementia, psychiatric symptoms

Alzheimer's disease (AD) is a debilitating neurodegenerative disorder and the most common cause of dementia (1). It was first described in 1906 by the German physician, Alois Alzheimer, who reported for the first time the presence of anatomical alterations in the brain of a patient with symptoms of dementia, describing it as a peculiar and severe disease process of the cerebral cortex (2). Characterized by the progressive loss of neurons and the dysfunction of several cognitive abilities including memory and learning, AD imposes a severe burden to the affected individuals, but also to their caregivers and to the society in terms of health care costs (3). Globally, more than 50 million individuals are affected with AD or other forms of dementia, and this number is estimated to rise above 130 million by the year 2050 (7). AD is particularly frequent in the elderly population and is one of the leading causes of disability in everyday functioning. Although its greatest risk factor is increasing age, other factors such as genetic mutations, mitochondrial dysfunction, inflammation, and malnutrition can increase the likelihood of developing AD (4).

For several decades, the Amyloid Cascade Hypothesis has been the prevailing concept underlying AD research (5). This hypothesis posits that the accumulation of the peptide amyloid-beta (Aβ) is the main cause of the condition (5). A series of events subsequently takes place following the brain accumulation of Aβ, including tau protein hyperphosphorylation and the formation of neurofibrillary tangles inside nerve cell bodies (5). Recent evidence has also shown that, beside cognitive deterioration, the vast majority of AD patients develop psychiatric symptoms such as depression, apathy, hallucinations and delusions at some stage during their disease, thus further worsening clinical outcome (6). Accordingly, over the past few years, several investigators have explored the possibility of treating the psychiatric symptoms in AD in an attempt to halt the progression of the disease. However, despite significant progress in research, reliable treatments for AD have not been found and currently prescribed medications are only symptomatic in nature. Further understanding of the neurobiological underpinnings of psychiatric symptoms in AD is necessary as this may pave the way toward the development of more efficient and integrated treatments.

The present Research Topic aims at providing a collection of articles delineating the psychiatric symptoms in AD and discussing the incorporation of these symptoms into revised diagnostic criteria and therapeutic guidelines. It includes 3 original research articles, one case report, and one perspective paper.

In the study by Wilczyńska et al. the authors investigate the usefulness of serum Aβ, tau protein (t-tau), and the recently discovered marker, YKL-40, in the detection and differentiation of several types of dementia. Their study employed 60 individuals with dementia, with either AD, vascular dementia, or mixed dementia, as well as 20 cognitively normal subjects over the age of 60. Using the enzyme-linked immunosorbent assay (ELISA) to measure the serum concentration of the different AD-related proteins, the authors of the study showed that YKL-40 may serve as a sensitive and specific biomarker of early dementia and, to a lesser extent, of dementia progression.

In the original paper by Wiels et al. the authors analyzed a memory clinic-based research database of 476 individuals with mild cognitive impairment (MCI) and 978 individuals with AD. Results of their study show that MCI is commonly associated with affective and sleep-related disorders, and that the severity of AD is correlated with the occurrence of depressive symptoms. Interestingly, the authors reported no significant differences in neuropsychiatric symptoms when comparing “pure” AD to AD with a significant vascular component, suggesting that the cerebrovascular complications in AD do not exacerbate neuropsychiatric symptoms.

The study by Senczyszyn et al. investigated the effect of computerized cognitive training (CCT) combined with whole body stimulation (WBC) on cognitive functions in individuals with MCI. Study participants included 84 adults aged 60 or older. Measurements of cognitive functions and depressive symptoms were evaluated with the Montreal Cognitive Assessment (MoCA) and Geriatric Depression Scale (GDS), respectively. Their findings indicate that CCT, especially in combination with WBC, significantly ameliorates several cognitive functions, including learning and memory, and improves depression-like behaviors. The authors of the study suggest that such strategy could serve as an effective treatment modality for improving cognitive performance and depression-like behaviors.

The present special issue also includes a case report by Kanamori et al. that describes for the first time the successful use of trazodone and fluvoxamine for the treatment of pica, defined as the compulsive eating of non-nutritive substances, in a 80-year old woman with AD.

Finally, the perspective study by Muñiz et al. highlights the importance of using a syndromic approach for optimizing psychotropic treatment in individuals with dementia. In particular, they state that one of the main medical reasons of drugs over- and miss-prescriptions is symptom-based prescription. By switching to syndrome-based prescription, a large proportion of drugs could be de-prescribed and some re-adjusted or kept.

## Author Contributions

All authors contributed to the writing of the manuscript.

## Conflict of Interest

The authors declare that the research was conducted in the absence of any commercial or financial relationships that could be construed as a potential conflict of interest.

## Publisher's Note

All claims expressed in this article are solely those of the authors and do not necessarily represent those of their affiliated organizations, or those of the publisher, the editors and the reviewers. Any product that may be evaluated in this article, or claim that may be made by its manufacturer, is not guaranteed or endorsed by the publisher.
